# Parent engagement and attendance in PEACH™ QLD – an up-scaled parent-led childhood obesity program

**DOI:** 10.1186/s12889-017-4466-6

**Published:** 2017-06-09

**Authors:** Susan L. Williams, Wendy Van Lippevelde, Anthea Magarey, Carly J. Moores, Debbie Croyden, Emma Esdaile, Lynne Daniels

**Affiliations:** 1 Central Queensland University, School of Health, Medical and Applied Sciences, Building 6, Bruce Highway, Rockhampton, QLD 4702 Australia; 2 Department of Public Health, Ghent University, De Pintelaan 185 – 4K3 room 036, 9000 Ghent, Belgium; 3 Flinders University, Nutrition and Dietetics, School of Health Sciences, Faculty of Medicine, Nursing and Health Sciences, Sturt Road, Bedford Park, Adelaide, SA 5042 Australia; 4 Queensland University of Technology, School of Exercise and Nutrition Sciences, Faculty of Health, Centre for Children’s Health Research (CCHR), Level 6, 62 Graham St, South Brisbane, Qld 4101 Australia

**Keywords:** Childhood obesity, Engagement, Enrolment, Attendance, Up-scaled, Treatment programs

## Abstract

**Background:**

Parenting, Eating and Activity for Child Health (PEACH™) is a multicomponent treatment program delivered over ten group sessions to parents of overweight/obese primary school-aged children. It has been shown to be efficacious in an RCT and was recently translated to a large-scale community intervention funded by the Queensland (Australia) Government. Engagement (enrolment and attendance) was critical to achieving program outcomes and was challenging. The purpose of the present study was to examine sample characteristics and mediating factors that potentially influenced program attendance.

**Methods:**

Data collected from parents who attended at least one PEACH™ Queensland session delivered between October 2013 and October 2015 (47 programs implemented in 29 discrete sites), was used in preliminary descriptive analyses of sample characteristics and multilevel single linear regression analyses. Mediation analysis examined associations between socio-demographic and parent characteristics and attendance at group sessions and potential mediation by child and parent factors.

**Results:**

365/467 (78%) enrolled families (92% mothers) including 411/519 (79%) children (55% girls, mean age 9 ± 2 years) attended at least one session (mean 5.6 ± 3.2). A majority of families (69%) self-referred to the program. Program attendance was greater in: advantaged (5.9 ± 3.1 sessions) vs disadvantaged families (5.4 ± 3.4 sessions) (*p* < 0.05); partnered (6.1 ± 3.1 sessions) vs un-partnered parents (5.0 ± 3.1 sessions) (*p* < 0.01); higher educated (6.1 ± 3.0 sessions) vs lower educated parents (5.1 ± 3.3 sessions) (*p* = 0.02); and self-referral (6.1 ± 3.1) vs professional referral (4.7 ± 3.3) (*p* < 0.001). Child (age, gender, pre-program healthy eating) and parent (perceptions of child weight, self-efficacy) factors did not mediate these relationships.

**Conclusions:**

To promote reach and effectiveness of up-scaled programs, it is important to identify ways to engage less advantaged families who carry higher child obesity risk. Understanding differences in referral source and parent readiness for change may assist in tailoring program content. The influence of program-level factors (e.g. facilitator and setting characteristics) should be investigated as possible alternative mediators to program engagement.

## Background

Childhood overweight is common and has important short and long-term adverse health outcomes [1]. In recent years, several child weight management interventions with demonstrated efficacy in the research setting have been translated and up-scaled into real-world settings [2, 3] in an effort to benefit more people and to foster policy and sustainable program development [4]. A key challenge of scaling-up is ensuring a program reaches a substantial and representative proportion of the eligible population whilst retaining effectiveness, requiring consideration of a broad range of factors that promote effectiveness, reach and adoption of a program [5]. When scaling-up a child weight management program this includes effective engagement (recruitment and attendance) of parents and children.

Evidence from randomized controlled trials and treatment programs (including up-scaled programs) for childhood obesity have identified a range of factors associated with engagement (recruitment and attendance) of parents and children. At the family level, factors inversely associated with engagement include parent socio-demographic characteristics: social disadvantage [3, 6–9], single/lone parenting [9], family income [7, 10]; parental confidence/parenting self-efficacy [11]; parents’ own nutrition and physical activity behaviors and stage of change for lifestyle behaviors [12] and child factors: age [6, 13], gender (boys) [3, 9], depressive symptoms/psychological distress [6, 9, 10], and weight status [8, 9, 11, 12]. At the program level, there is evidence of inverse associations between engagement and relevance of the program, travel distance to a program site; timing of program sessions (weekends compared to weekdays) [10]; and size of group and/or facilitator familiarity in delivery of a program [9].

Qualitative studies have found both parent and child factors associated with program engagement. Program enrolment is enhanced by a parent’s awareness of their child’s overweight status and desire to improve their child’s health [14, 15]. Program attendance is improved by children’s involvement in attendance decisions [16]; and program completion driven by a child’s development during a program, of social groups and improvement in their self-esteem and confidence [14, 16]. Identified barriers to program engagement include: delivery of a program in a clinical environment [16, 17]; mismatch in pre-conceived perceptions of the intervention [15]; scheduling conflicts [18]; lack of family support [14, 18]; transportation barriers; and unmet expectations [15–18].

Recruitment processes have also been found to impact engagement in child overweight treatment programs. Active/professional referrals and passive/self-referrals (that rely on public advertising and word-of-mouth) are commonly used, either alone or in combination [19]. In some cases, professional referrals have provided an efficient and effective pathway to enrolment in a program (i.e. in relation to numbers enrolled and cost) [19–22] however, the overall attendance of families professionally referred has been shown to be less when compared to those who are self-referred [19, 21].

Although factors related to parent/family engagement are well documented, successful enrolment, attendance, participation and completion of a program, remains challenging [23–26]. When scaling up, any failure to get participants to enrol and attend, can limit the capacity for outcome evaluation to demonstrate effectiveness [27] and can also have consequences on program cost-effectiveness, adoption and sustainabilty. Investigating the mediating factors of program engagement can provide insight into mechanisms underlying successful engagement with the program and potential aspects of systems and processes that could/should be improved [28]. Thus, the aim of this analysis was to assess predictors of parent attendance in an up-scaled efficacious intervention program to treat childhood obesity. Specifically, we examined associations between referral sources and parent socio-demographic factors and attendance at parent group sessions, and conducted exploratory analyses of causal mechanisms of attendance through mediation analysis.

## Methods

Parenting, Eating and Activity for Child Health (PEACH™) is a family-based lifestyle intervention to treat overweight primary school aged children [29] which targets parents as the agents of change [30] and is thus, delivered via group sessions to parents, rather than children. It has been demonstrated to be efficacious in a randomized controlled trial with children achieving a relative weight loss of ~10% [29] that was maintained for 18 months post-intervention. The Queensland Government funded the state-wide implementation of the PEACH™ program between 2013 and 2016. Eligibility criteria required families to be residing in areas of Queensland where the program was being offered, and have children aged 5–11 years who were overweight/obese at the time of enrolment (IOTF cut-point) [31].

The original PEACH™ intervention delivered ten sessions spread over a six month period. However, after initial delivery of the program in Queensland (*n* = 229 families; 251 children) and in response to parent and facilitator feedback and a range of feasibility issues, modifications were made to the delivery schedule to fit nine sessions within a school term with a final session scheduled to retain an overall follow-up period of six months. The PEACH™ QLD program subsequently included nine 90 min face-to-face sessions delivered weekly over a school term with a tenth final ‘review and measurement’ session at around six months. Sessions were scheduled outside school hours and delivered in a range of community settings, tertiary hospitals, schools and universities. The parent sessions were facilitated by project-funded health professionals, most commonly dietitians and nutritionists, who received standardized training. Enrolled children (and sometimes their siblings) participated in concurrent and separate child sessions (90 min) which included standardized non-competitive games and physical activities.

Ethics approvals for this study were provided by Queensland Children’s Health Services Human Research Ethics Committee (EC00175) (Project reference HREC/13/QHC/25) on 17 September 2013; Queensland University of Technology University Human Research Ethics Committee (EC00171) (Project reference 1,300,000,633) on 23 October 2013; Flinders University Social and Behavioural Research Ethics Committee (EC00194) (Project reference 6231) on 18 September 2013; and Central Queensland University Human Research Ethics Committee (EC00158) (Project reference H13/09-173) on 3 October 2013. All ethics applications were submitted with the National Ethics Application Form (AU/1/D1F2110) lodged 3 June 2013.

### Recruitment

Recruitment occurred through either: (i) self-referral: in response to extensive promotion via media (print, radio, television), schools and social media with parents registering their interest via the program website or toll free number; or (ii) professional referral: health professional or hospital waiting lists. All potential participants were telephoned by study staff to assess eligibility and provide information on program format, venue, and timing. Eligible families who agreed to enrol were forwarded (by email or post as requested) a confirmation of enrolment letter and welcome pack, which included detailed information pertaining to their closest group, consent forms, information sheet, postcard welcoming the child/ren to the program and a web link to the online baseline evaluation questionnaire (Survey Monkey Inc., Palo Alto, California, USA).

### Data collection

The primary carer of the enrolled child/ren was requested to complete a baseline evaluation questionnaire which provided data about family socio-demographic characteristics, referral source (independent variables) and parent perceptions of child health, parenting self-efficacy, child age, gender and healthy eating (mediating factors). These were completed on-line prior to attendance at the first session or in paper format at the first session.

### Independent variables

**Socio-demographic characteristics** used were: *marital status* (partnered versus un-partnered); *education attainment* (year 12 or less versus technical and further education (TAFE/trade certificate/University degree), and residential postcode. Postcodes were used to derive *Socioeconomic Indexes for Areas (SEIFA) Index of Relative Socioeconomic Advantage and Disadvantage (IRSAD)*, compiled by the Australian Government and based on 2011 Census data [32]. For analysis, the IRSAD deciles (1 = most disadvantaged, 10 = most advantaged) were dichotomized into: (i) most advantaged (deciles 5–10); or (ii) most disadvantaged (deciles 1–4) [33]. *Referral Source* was defined as professional versus self-referral as described above.

### Mediating factors

**Parent perception of child health** was assessed via two 5-point Likert-scale questions based on the Health Belief Model [34]: (i) *Perceived severity of weight problem in child* (Do you think that your child’s weight is a serious health concern?); and (ii) *Perceived susceptibility of child to chronic disease* (Do you think your child’s weight increases their risk of developing other illnesses?). Response options were 1 = Not serious to 5 = Very serious.

**Parent confidence and parenting self-efficacy** were measured by seven items representing two constructs: *(1) Parental confidence to create a healthy home environment* consisted of three items: How confident do you feel about: (i) making healthy changes to your child’s/family’s eating and activity patterns?; (ii) setting limits regarding your child’s food and eating?; and (iii) setting limits regarding your child’s activity/inactivity patterns? Response options ranged from 1 = Not at all confident to 5 = Extremely confident, and a mean score based on the three items was derived; (2) Four items from the Longitudinal Study of Australian Children (LSAC) [35] relating to *General parenting self-efficacy* asked parents: (i) Does your child behave in a manner different from the way you want him/her to?; (ii) Do you think that your child’s behavior is more than you can handle?; (iii) Do you feel that you are good at getting this child to do what you want him/her to do?; and (iv) Do you feel that you are in control and on top of things when you are caring for your child? Response options ranged from 1 = Never/almost never to 5 = Almost always/always). Items (i) and (ii) were reverse scored and a mean of the four items was calculated such that a higher score indicated greater parenting self-efficacy.

**Child Healthy Eating** was assessed from parent-reported, child average intakes (on school days and weekend days) of the number of serves of each of the core food groups (vegetables; fruit; grains (cereal) foods; lean meats and poultry, fish, eggs, tofu, nuts and seeds, and legumes/beans; milk, yoghurt, cheese and/or alternatives [36]. Illustrations depicting examples of serve sizes were provided as a guide to assist with serve size estimations. Number of serves of each food group/day were calculated and age and gender cut-offs applied in accordance with the Australian Guide to Healthy Eating [37] for: meeting (=1) or not meeting (=0) recommendations for each food group. The construct *Child healthy eating* was created by summing these five variables indicating adherence to the core food group recommendations [0–5] with a high score (5) indicating healthier eating.

### Outcome variable

Attendance at each session was recorded via parent sign-in sheets. The outcome (dependent) variable was defined as the total number of sessions attended by each parent (1–10). Data are only included from parents who attended at least one session as the majority who did not attend any sessions did not provide consent or baseline data. Data from second and third enrolled children (*n* = 53) of families with more than one child enrolled (*n* = 27) were not included in analysis.

## Data analysis

### Statistical analyses

Data from 47 groups conducted in 29 discrete sites over two years from October 2013 were utilized. Variables that had a significant association with attendance were utilized in the regression and mediation analyses. These were the independent variables (marital status; socioeconomic status according to education attainment; socioeconomic status according to SEIFA IRSAD; referral source) and mediating factors (parent-perceived severity of weight problem in child and susceptibility of child to chronic disease; parent confidence to create a healthy home environment; general parenting self-efficacy; child age, gender and healthy eating). Descriptive analysis was undertaken using SPSS 21.0. Multilevel single linear regression analyses (2-level: parents in intervention groups) were conducted using MLwiN version 2.30.

**Fig. 1:**
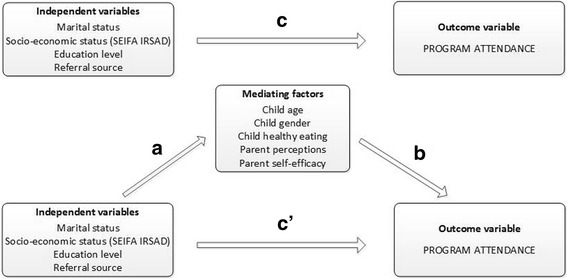
Conceptual mediation model. Mediation model of the relationship between socio-demographic factors/referral source and attendance as mediated through child (age, gender, healthy eating) and parent factors (perceptions of child health; and self-efficacy (confidence to create healthy home environment and general parenting)

For the mediation analyses (as represented in Fig. 1) the product-of-coefficient test of MacKinnon and colleagues was used [28, 38]. The first step was to investigate the difference in attendance according to each independent variable (c-path). The second step was to estimate the difference in the potential mediator according to the independent variable (action theory test: a-coefficient). The third step in the mediation model was to estimate (1) the difference in attendance (=the dependent variable) according to the potential mediators (=child age, child healthy eating) and controlled for the independent variables (=marital status, parent education, socio-economic status) (conceptual theory test: b-coefficient); and (2) the difference in attendance according to the independent variable controlled for the potential mediator (c’-path). To represent the mediated effect, the product of the two coefficients (a coefficient*b coefficient), was calculated [38]. The statistical significance of the mediated effect was estimated by dividing the product-of-coefficient (a*b) by its standard error and then compared to a standard normal distribution. For the calculation of the standard error the Sobel formula was used (SE_ab_ = √(a^2^*SE_b_^2^+b^2^*SE_a_^2^). Statistical significance was set at the *p* < 0.05 level.

## Results

### Sample characteristics

Of the 467 families enrolling 365 (78%) families (92% mothers) including 411/519 (79%) children (55% girls; mean age 9 ± 2 years) attended at least one session and provided baseline data. A total of 28 parents (1 father, 27 mothers, all were biological parents) who did not attend any session provided baseline data. Follow-up phone calls with non-attendees were attempted by program facilitators at the time of non-attendance however most parents could not be contacted or did not provide specific reasons for non-attendance. Characteristics of parents who attended at least one session are shown in Table 1. These parents attended a mean of 5.6 ± 3.2 sessions and 11% attended all ten sessions. Sixty-nine percent of families self-referred to the program. The mean number of healthy eating recommendations met by children was 1.2 ± 1.2 (range 1–5) and 25.5% did not meet any recommendations. Pearson chi-square analyses of differences between parents who attended only one session and parents who attended all ten sessions showed that parents attending all ten sessions were more educated (*p* = 0.008), less disadvantaged (*p* = 0.03) and self-referred to the program (*p* = 0.03).

**Table 1:** Characteristics of parents attending at least one session of the PEACH™ QLD program

Characteristic	*n* = 338^a^ *n* (%)
**Parent sociodemographic factors**	
*Marital status* (Partnered; Unpartnered)	
Partnered	239 (71)
*Parent education* (Year 12 or less; TAFE/trade/University degree)	
12 years of schooling or less	113 (34)
*Socioeconomic status* (SEIFA IRSAD^c^)	
Most disadvantaged (deciles 1–4)	97 (29)
**Parent mediating factors** ^**d**^	**Mean ± SD** ^**b**^
*Parent perceptions*	
Severity of weight problem in child (1–5)	3.4 ± 1.2
Susceptibility of child to chronic disease (1–5)	3.5 ± 1.2
*Parent self-efficacy*	
Confidence to create healthy home environment (1–5)	3.0 ± 0.9
General parenting (1–5)	3.5 ± 0.8

^a^Second and third children of families with more than one child enrolled in the program, were not included in analyses

^b^Standard Deviation

^c^Socio-Economic Indexes for Areas (SEIFA) Index of Relative Socioeconomic Advantage and Disadvantage [32]

^d^Higher scores represent greater concern or efficacy

### Associations between socio-demographic and referral source and program attendance (c-path Fig. 1)

Program attendance was influenced by marital status, socioeconomic status (SES) according to both SEIFA and educational level, and referral source (Table 2). Program attendance was greater in advantaged versus disadvantaged families (5.9 ± 3.1 vs 5.4 ± 3.4 sessions; *p* < 0.05); partnered versus un-partnered parents (6.1 ± 3.1 vs 5.0 ± 3.1 sessions; *p* < 0.01); higher educated versus lower educated parents (6.1 ± 3.0 vs 5.1 ± 3.3 sessions; *p* = 0.02) and families who self-referred versus those professionally referred (6.1 ± 3.1 vs 4.7 ± 3.3 sessions; *p* < 0.001).

**Table 2:** Unadjusted multilevel linear regression coefficients (95% CI) of associations between independent variables and sessions attended^a^

Independent variables	c (SE)	*p*	95% CI
Marital status (ref = Partnered)	**−1.058 (0.366)**	**0.004**	−1.775;-0.341
SES according to SEIFA IRSAD (ref = Most disadvantaged)	**0.821 (0.399)**	**0.04**	0.039;1.603
SES according to education attainment (ref = 12 years of schooling or less)	**0.912 (0.353)**	**0.01**	0.130;1.694
Referral source (ref = Self referred)	**−1.309 (0.376)**	**0.005**	−2.046;-0.572

^a^Sessions attended by parents (*N* = 338) of 5–11 year old children classified overweight/obese as defined by International Obesity Taskforce (IOTF) cut points [31]

*c* coefficients, *SE* standard error, *CI* Confidence Interval; ref. = reference category

SEIFA IRSAD - Socio-Economic Indexes for Areas (SEIFA) Index of Relative Socioeconomic Advantage and Disadvantage [32]

All significant associations are presented in **bold** font

### Associations between socio-demographic factors and referral source, and potential mediators (path a Fig. 1)

As shown in Table 3 (action theory tests), in higher educated families, children had healthier eating (as defined in methods) than those from lower educated families. Parents who were professionally referred more often had a boy enrolled in the program, and had greater concern for their child’s weight status and its consequences, compared to the self-referred parents.

**Table 3:** Mediating role of child and parent factors on associations between socio-demographic characteristics, referral source, and attendance^a^

Single mediation models	Action theory tests^a^	*p*	Conceptual theory tests^b^	*p*	Mediating effects
	*a* (SE)		*b* (SE)		*ab* (SE)
Marital status (Partnered, Un-partnered)					
Child age	0.205 (0.211)		**−0.208 (0.095)**	0.028	−0.043 (0.048)
Child gender	−0.03 (0.059)		0.161 (0.335)		−0.005 (0.014)
Child healthy eating^b^	−0.098 (0.135)		**0.402 (0.152)**	0.008	−0.039 (0.056)
Parent perception^c^: severity of weight problem in child	0.207 (0.140)		0.013 (0.142)		0.003 (0.029)
Parent perception^c^ susceptibility of child to chronic disease	0.155 (0.147)		0.179 (0.135)		0.028 (0.034)
Parent confidence^c^ in creating healthy home environment	0.037 (0.103)		−0.186 (0.193)		−0.007 (0.020)
Parent self-efficacy^c^ for general parenting	−0.046 (0.099)		−0.044 (0.202)		0.002 (0.010)
SES according to SEIFA IRSAD (Most advantaged, Most disadvantaged)					
Child age	0.070 (0.213)		**−0.21 (0.096)**	0.03	−0.015 (0.045)
Chid gender	−0.002 (0.060)		0.198 (0.337)		0.000 (0.012)
Child healthy eating^b^	−0.118 (0.136)		**0.424 (0.153)**	0.006	−0.050 (0.060)
Parent perception^c^: severity of weight problem in child	0.007 (0.143)		−0.006 (0.143)		0.000 (0.001)
Parent perception^c^: susceptibility of child to chronic disease	−0.002 (0.152)		0.158 (0.136)		0.000 (0.024)
Parent confidence^c^ to create healthy home environment	−0.024 (0.105)		−0.184 (0.194)		0.004 (0.020)
Parent self-efficacy^c^ for general parenting	0.083 (0.104)		−0.054 (0.204)		−0.004 (0.018)
SES according to education attainment (Year 12 or less schooling, TAFE/trade/University Degree)					
Child age	0.003 (0.204)		**−0.224 (0.095)**	0.02	−0.001 (0.046)
Child gender	0.051 (0.057)		0.151 (0.336)		0.008 (0.019)
Child healthy eating^b^	**0.262 (0.129)**	0.04	**0.388 (0.154)**	0.01	0.102 (0.064)
Parent perception^c^: severity of weight problem in child	−0.168 (0.135)		0.007 (0.143)		−0.001 (0.024)
Parent perception^c^: susceptibility of child to chronic disease	−0.108 (0.142)		0.175 (0.136)		−0.019 (0.029)
Parent confidence^c^ to create healthy home environment	−0.147 (0.099)		−0.161 (0.194)		0.024 (0.033)
Parent self-efficacy^c^ for general parenting	0.036 (0.095)		−0.043 (0.203)		−0.002 (0.008)
Referral source (Professional, Self-referred)					
Child age	0.258 (0.205)		**−0.253 (0.094)**	0.007	−0.065 (0.057)
Child gender	**−0.129 (0.057)**	0.02	0.304 (0.330)		−0.039 (0.046)
Child healthy eating^b^	0.083 (0.139)		**0.464 (0.154)**	0.003	0.039 (0.066)
Parent perception^c^: severity of weight problem in child	**0.391 (0.143)**	0.006	0.055 (0.144)		0.022 (0.057)
Parent perception^c^: susceptibility of child to chronic disease	**0.429 (0.149)**	0.004	0.216 (0.137)		0.093 (0.067)
Parent confidence^c^ to create a healthy home environment	0.213 (0.105)		−0.138 (0.194)		−0.029 (0.044)
Parent self-efficacy^c^ for general parenting	−0.014 (0.104)		0.000 (0.204)		0.000 (0.003)

^a^Attendance at the PEACH™ QLD program by parents (*n* = 338) of 5–11 year old children classified overweight/obese as defined by International Obesity Taskforce (IOTF) cut points [31]

a-coefficient: estimate (regression coefficient) of the difference in the mediator by the independent variable

b-coefficient: estimate (regression coefficient) of the difference in attendance related to the mediator and adjusted for the total effect of the independent and outcome variable

ab product-of-coefficient estimate; mediated effect

Two-level single regression models were conducted: parents within intervention groups

^b^Child healthy eating (as defined in methods) – healthier eating

^c^Higher concern or efficacy

All significant associations are presented in **bold** font

### Associations between potential mediators and program attendance (path b Fig. 1)

As shown in Table 3 (conceptual theory tests), for all socio-demographic and referral source variables, children’s age and healthy eating at baseline was associated with attendance. The younger the child and healthier their eating, the higher the attendance of the family.

### Mediation effects (ab product)

Results of mediation analysis are shown in Table 3. None of the examined family- and child-related factors showed a mediating effect on the relation between the socio-demographic factors and referral source and program attendance.

## Discussion

The purpose of the present study was to examine sample characteristics and mediating factors that potentially influence parent attendance at group sessions that delivered an up-scaled treatment intervention for overweight/obese primary school age children. Child age, gender and pre-program healthy eating pattern nor, parent perception of child weight and self-efficacy mediated the associations found between attendance and parent education, social advantage, partner status and self-referral. Our lack of significant mediating effects suggests that the expected association of program attendance with both relative advantage and referral source are not explained by the child factors (age, gender or pre-program diet quality) or parenting factors (self-efficacy related to provide a healthy home environment and general parenting; concern regarding their child’s weight status and consequences) included in this analysis.

Families who attended at least one session overall attended almost 60% of sessions but only 1:10 families attended all ten sessions. Other scaled-up programs such as: the Mind, Exercise, Nutrition, Do it! (MEND) program (conducted in England between 2007 and 2010 [9]) and Go4Fun® (an Australian version of MEND conducted in New South Wales, Australia between 2009 and 2012 [3]), have reported that 59% and 58% of their families attended at least 75% of 20 sessions, respectively. Comparing attendance and attrition rates across studies however is difficult, as these variables are expressed and reported in a range of ways. Program characteristics such as frequency; number of sessions and duration; and group versus one-to-one contact, also vary. Two recent reviews report attrition/drop-out rates from pediatric weight management interventions between 25 and 75% and highlight significant differences in engagement in relation to study design (RCT 0–42%) versus clinical (up to 64%) [39], SES and ethnicity [40], and also discordance between family needs/expectations, and program scheduling and content [39].

Of great concern in this current study, and also reported for the MEND program [9], is that approximately 23% of families who completed screening and enrolled for the program failed to attend even one session and/or provide pre-evaluation data (data not shown). Non-attendance may relate to reasons identified in previous qualitative studies (for example, scheduling conflicts, travel and timing [10, 18] however conclusions cannot be made without adequate follow-up data from these families. Although, follow-up phone and/or email contact with all non-attending parents was attempted by session facilitators after conduct of session one, reasons for non-attendance and consent to use of data were not obtained from an adequate number of non-attending parents. For future up-scaled programs, it will be important to commit more resources to conducting follow-up with non-attending families to ensure reasons for non-attendance are captured and modifications to a program can subsequently be considered.

We found that parents who were single/un-partnered, with lower education attainment and greater levels of social disadvantage had lower program attendance rates, a finding similar to earlier reports [6, 7, 9]. In agreement with findings from other similar programs, we also found that those who were self-referred had better program attendance [19, 21]. However, none of our hypothesized child and parent factors were shown to mediate these relationships. Thus, there remains a need to identify modifiable mediating factors that have potential to ameliorate the impact of disadvantage on attendance. It is important to note that overall, advantaged families attended approximately one additional session. Understanding the impact of differences in attendance on overall program outcomes is problematic as most participants who actively or passively withdraw (do not attend) do not provide outcome data.

Results of our regression analyses provide further understanding of some factors related to attendance. Parents had better attendance when their child was younger or their child had healthier eating prior to the program. These findings align with previous studies that report greater attendance in families with younger children [6, 13] and in parents who may be ready to make changes around child eating and physical activity behaviors and more commonly in the action stage of change for dietary behaviors (compared to physical activity) [41]. It is conceivable that parents may be less ready or confident to change existing habits in older children than younger children. Additionally, it is possible that parents of children already eating comparatively well felt affirmed and encouraged by the program and hence were more likely to maintain their engagement.

Previous qualitative studies have reported enhanced program enrolment by parents with greater awareness of their child’s overweight status [14, 15]. Although, we found that professionally referred parents had higher levels of concern for their child’s weight status and its consequences on child health, these families attended on average 1.5 sessions less than those who were self-referred. Plausible explanations that align with previous studies [19, 21] include that self-referred parents were seeking support and were therefore more committed to the program and for professionally referred families, their enrolment in the program was not based on personal motivations (and/or their readiness to change) and/or their decision to attend did not involve their children, subsequently limiting their ongoing commitment to program attendance [16]. Another possible explanation based on the historical management of child obesity (i.e. one: one clinical consultations with parent and child) is that parents who were professionally referred may have had preconceived perceptions and expectations around their child’s treatment requirements that conflicted with the program content and approach, thereby impacting their behavioral decisions [15, 17, 18].

Our finding that parents referred by a health professional more often had a son enrolled in the program (in contrast to the self-referred parents that were more often seeking help for a daughter) aligns with other studies [42, 43] that have found parents more likely to identify their daughters as overweight than their sons. Hence, parents with sons may be more likely to require a clinician to recognize the issue and make a referral. Overall, the relationship between referral source and attendance has implications for the future delivery of publically-funded programs that may rely on clinician referrals to support funding applications. As a majority of our completing families self-referred to the program, future development of policies aimed at increasing access to publicly-funded child obesity treatment services and programs, should include this self-referral pathway to enhance reach and engagement and deliver a better return on investment.

A few limitations of the current analysis need to be mentioned. First, the included mediation variables were based on parent-reported data with the potential for responses to be socially desirable, although evaluation was largely completed independent of the group facilitator. The sample size included in this study may have limited the power of the mediation analyses. No Bonferroni correction was applied to control the overall type I error rate in these multiple significance tests. However, this correction was not applied as previous research has highlighted that this correction tends to be too strict when multiple tests are performed [44].

Overall, our findings show that, consistent with other programs, attendance was a challenge with very few families attending all planned sessions. Although there was the expected differential in attendance according to disadvantage and referral source, these associations were not explained by parent concern regarding their child’s weight status or their parenting self-efficacy and confidence to manage behavior change necessary to improve child and family eating and activity patterns. As such, pre-program screening for these factors to identify families who may need additional content or support is not likely to be effective in improving attendance. Other program-level factors (e.g. group size, competence of facilitator) may be important. Strategies such as the use of online technologies for flexible program delivery or incorporation of the program into school-based activities require consideration. An important question which remains is the extent to which poor attendance dilutes the effectiveness of the program in terms of lifestyle and weight status behavior changes and whether there is an overall or individual family critical attendance threshold. This question is very difficult to answer as those with poor attendance rarely provide useable final outcome data.

## Conclusions

Our experiences of family engagement in scaling up the PEACH™ program reflect the ongoing difficulties in providing treatment options for child overweight (irrespective of design or delivery methods) and are aligned with many previously reported studies. It is important that future up-scaled programs include development of comprehensive evaluation processes that permit extensive examination of factors that influence recruitment and attendance of parents and children. Specific considerations important to developing such programs include: identifying ways to enhance engagement of less advantaged families (who carry higher child obesity risk) in obesity treatment programs to enhance reach and effectiveness of up-scaled programs; understanding differences in referral source and parent readiness for change to enable tailoring of program content; and investigating the influence of program-level factors (e.g. facilitator and setting characteristics) as possible mediators to program engagement.

## Abbreviations

CI: Confidence Interval
HREC: Human Research Ethics Committee
IOTF: International Obesity Task Force
IRSAD: Index of Relative Socioeconomic Advantage and Disadvantage
LSAC: Longitudinal Study of Australian Children
MEND: Mind, Exercise, Nutrition, Do it!
PEACH™: Parenting, Eating and Activity for Child Health
QLD: Queensland
RCT: Randomized Controlled Trial
SD: Standard Deviation
SE: Standard Error
SEIFA: Socioeconomic Indexes for Areas
SES: Socioeconomic status
SPSS: Statistical Package of the Social Sciences
TAFE: Technical and Further Education
USA: United States of America

## References

[1] Han JC, Lawlor DA, Kimm SY (2010). Childhood obesity. Lancet (London, England).

[2] Fagg J, Chadwick P, Cole TJ, Cummins S, Goldstein H, Lewis H, et al. From trial to population: a study of a family-based community intervention for childhood overweight implemented at scale. Int J Obes (2005). 2014;38(10):1343–9. 10.1038/ijo.2014.103 PMC4175967 24919564

[3] Welsby D, Nguyen B, O'Hara BJ, Innes-Hughes C, Bauman A, Hardy LL (2014). Process evaluation of an up-scaled community based child obesity treatment program: NSW Go4Fun(R). BMC Public Health.

[4] WHO (2010). Nine steps for developing a scaling-up strategy.

[5] Milat AJ, King L, Bauman AE, Redman S (2013). The concept of scalability: increasing the scale and potential adoption of health promotion interventions into policy and practice. Health Promot Int.

[6] Zeller M, Kirk S, Claytor R, Khoury P, Grieme J, Santangelo M, et al. Predictors of attrition from a pediatric weight management program. J Pediatr. 2004;144(4):466–70. 10.1016/j.jpeds.2003.12.031 15069394

[7] Williams NA, Coday M, Somes G, Tylavsky FA, Richey PA, Hare M (2010). Risk factors for poor attendance in a family-based pediatric obesity intervention program for young children. J Dev Behav Pediatr.

[8] de Niet J, Timman R, Jongejan M, Passchier J, van den Akker E (2011). Predictors of participant dropout at various stages of a pediatric lifestyle program. Pediatrics.

[9] Fagg J, Cole TJ, Cummins S, Goldstein H, Morris S, Radley D, et al. After the RCT: who comes to a family-based intervention for childhood overweight or obesity when it is implemented at scale in the community? J Epidemiol Community Health. 2014; 10.1136/jech-2014-204155 PMC4316870 25294895

[10] Jensen CD, Aylward BS, Steele RG (2012). Predictors of attendance in a practical clinical trial of two pediatric weight management interventions. Obesity (Silver Spring, Md).

[11] Gunnarsdottir T, Njardvik U, Olafsdottir AS, Craighead LW, Bjarnason R (2011). The role of parental motivation in family-based treatment for childhood obesity. Obesity.

[12] Maximova K, Ambler KA, Rudko JN, Chui N, Ball GDC (2015). Ready, set, go! Motivation and lifestyle habits in parents of children referred for obesity management. Pediatr Obes.

[13] Danielsson P, Svensson V, Kowalski J, Nyberg G, Ekblom O, Marcus C (2012). Importance of age for 3-year continuous behavioral obesity treatment success and dropout rate. Obes Facts.

[14] Moore KG, Bailey JH (2013). Parental perspectives of a childhood obesity intervention in Mississippi: a Phenomological study. Qual rep.

[15] Newson L, Povey R, Casson A, Grogan S (2013). The experiences and understandings of obesity: families' decisions to attend a childhood obesity intervention. Psychol Health.

[16] Banks J, Cramer H, Sharp DJ, Shield JP, Turner KM (2014). Identifying families’ reasons for engaging or not engaging with childhood obesity services: a qualitative study. J Child Health Care.

[17] Kitscha CE, Brunet K, Farmer A, Mager DR (2009). Reasons for non-return to a pediatric weight management program. Can J Diet Pract res.

[18] Sallinen BJ, Schaffer S, Woolford SJ (2013). In their own words: learning from families attending a multidisciplinary pediatric weight management program at the YMCA. Child Obes (Print).

[19] Fleming J, Kamal A, Harrison E, Hamborg T, Stewart-Brown S, Thorogood M (2015). Evaluation of recruitment methods for a trial targeting childhood obesity: families for health randomised controlled trial. Trials..

[20] Rice J, Thombs D, Leach R, Rehm R (2008). Successes and barriers for a youth weight-management program. Clin Pediatr (Phila).

[21] Raynor HA, Osterholt KM, Hart CN, Jelalian E, Vivier P, Wing RR (2009). Evaluation of active and passive recruitment methods used in randomized controlled trials targeting pediatric obesity. Int J Pediatr Obes.

[22] Perez AJ, Avis JLS, Holt NL, Gokiert R, Chanoine JP, Legault L (2016). Why do families enrol in paediatric weight management? A parental perspective of reasons and facilitators. Child Care Health dev.

[23] Robertson W, Friede T, Blissett J, Rudolf MC, Wallis M, Stewart-Brown S (2008). Pilot of "families for health": community-based family intervention for obesity. Arch Dis Child.

[24] Watson PM, Dugdill L, Pickering K, Bostock S, Hargreaves J, Staniford L (2011). A whole family approach to childhood obesity management (GOALS): relationship between adult and child BMI change. Ann Hum Biol.

[25] Smith LR, Chadwick P, Radley D, Kolotourou M, Gammon CS, Rosborough J, et al. Assessing the short-term outcomes of a community-based intervention for overweight and obese children: the MEND 5-7 programme. BMJ Open. 2013;3(5). 10.1136/bmjopen-2013-002607 PMC3646180 23645925

[26] Gerards SM, Dagnelie PC, Gubbels JS, van Buuren S, Hamers FJ, Jansen MW (2015). The effectiveness of lifestyle triple P in the Netherlands: a randomized controlled trial. PLoS One.

[27] Milat A, Newson R, King L (2014). Centre for epidemiology and evidence. Increasing the scale of population health interventions: a guide.

[28] MacKinnon D (2008). Introduction to statistical mediation analysis.

[29] Magarey AM, Perry RA, Baur LA, Steinbeck KS, Sawyer M, Hills AP, et al. A parent-led family-focused treatment program for overweight children aged 5 to 9 years: the PEACH RCT. Pediatrics. 2011;127(2):214–22. 10.1542/peds.2009-1432 21262890

[30] Golan M (2006). Parents as agents of change in childhood obesity--from research to practice. Int J Pediatr Obes.

[31] Cole TJ, Bellizzi MC, Flegal KM, Dietz WH (2000). Establishing a standard definition for child overweight and obesity worldwide: international survey. BMJ.

[32] Socio-Economic Indexes for Areas. [http://www.abs.gov.au/websitedbs/censushome.nsf/home/seifa].

[33] Montgomerie AM, Chittleborough CR, Taylor AW (2014). Physical inactivity and incidence of obesity among South Australian adults. PLoS One.

[34] Rosenstock IM (1974). The health belief model and preventive health behavior. Health Educ Behav.

[35] The Longitudinal Study of Australian Children Annual Statistical Report 2010 [http://www.growingupinaustralia.gov.au/pubs/asr/2010/asr2010e.html].

[36] Golley RK, Hendrie GA, Slater A, Corsini N (2011). Interventions that involve parents to improve children's weight-related nutrition intake and activity patterns - what nutrition and activity targets and behaviour change techniques are associated with intervention effectiveness?. Obes rev.

[37] National Health and Medical Research Council NHMRC (2013). Australian dietary guidelines.

[38] MacKinnon DP, Luecken LJ (2008). How and for whom? Mediation and moderation in health psychology. Health Psychol.

[39] Skelton JA, Beech BM (2011). Attrition in paediatric weight management: a review of the literature and new directions. Obes rev.

[40] Ligthart KAM, Buitendijk L, Koes BW, van Middelkoop M. The association between ethnicity, socioeconomic status and compliance to pediatric weight-management interventions – a systematic review. Obes Res Clin Pract. 10.1016/j.orcp.2016.04.001 27108215

[41] Rhee KE, McEachern R, Jelalian E (2014). Parent readiness to change differs for overweight child dietary and physical activity behaviors. J Acad Nutr Diet.

[42] Jeffery AN, Voss LD, Metcalf BS, Alba S, Wilkin TJ (2005). Parents' awareness of overweight in themselves and their children: cross sectional study within a cohort (EarlyBird 21). BMJ (Clinical Research Ed).

[43] Campbell MW, Williams J, Hampton A, Wake M (2006). Maternal concern and perceptions of overweight in Australian preschool-aged children. Med J Aust.

[44] Field A (2013). Discovering Statistics Using IBM SPSS Statistics (4th ed).

